# Maximizing Single-Feature Separability for Improving Transfer Learning in Motor Imagery EEG Decoding

**DOI:** 10.3390/brainsci16020230

**Published:** 2026-02-14

**Authors:** Zefeng Xu, Zhuliang Yu

**Affiliations:** School of Automation Science and Engineering, South China University of Technology, Guangzhou 510641, China; auzefengxu@mail.scut.edu.cn

**Keywords:** motor imagery, EEG, transfer learning, feature separability, regularization

## Abstract

Background/Objectives: Motor imagery (MI) EEG-based brain–computer interfaces (BCIs) are promising for neurorehabilitation, but practical use is often hindered by time-consuming per-user calibration and performance instability across sessions/users. Methods: To mitigate this issue, we aim to improve subject-dependent MI classification by leveraging labeled training data from other subjects within the same dataset via transfer learning. We propose Maximizing Single-Feature Separability (MSFS), a lightweight plug-in regularization applied during target–subject fine-tuning. MSFS operates on the network feature layer and constructs batch-wise target positions by maximizing a silhouette-based separability criterion for each feature dimension. The target position computation is implemented in a fully vectorized GPU-friendly manner. Results: We evaluate MSFS on BCI Competition IV-2a and IV-2b datasets using three representative backbone networks (EEGNet, ShallowConvNet, ATCNet). MSFS consistently improves standard transfer learning across both datasets and all backbones. When compared against representative transfer learning algorithms from the literature, MSFS remains competitive against the literature baselines. Ablation analysis confirms the effectiveness of each algorithm component. Few-shot experiments further indicate that MSFS is still beneficial when the target subject provides limited labeled data. Conclusions: MSFS provides a within-dataset transfer learning enhancement for MI EEG decoding, improving target–subject accuracy under limited calibration data without relying on external datasets, and can be readily integrated into common deep MI classification pipelines.

## 1. Introduction

Brain–computer interfaces (BCIs) provide a direct pathway between neural activity and external devices, enabling communication and control without relying on peripheral nerves or muscles [1]. Among multiple BCI paradigms, electroencephalography (EEG)-based systems remain particularly attractive due to their noninvasiveness, portability, and high temporal resolution [2]. Motor imagery (MI) is one of the most widely studied EEG paradigms, where users voluntarily imagine limb movements (e.g., left/right hand) without actual movement execution. MI induces characteristic modulations of sensorimotor rhythms (SMRs), typically reflected by event-related desynchronization/synchronization (ERD/ERS) in the μ (8–13 Hz) and β (13–30 Hz) bands over sensorimotor cortices [2,3]. From a neurophysiological perspective, MI engages neural substrates partially overlapping with motor preparation and action execution, which helps explain why rhythmic power changes and spatial topographies over the sensorimotor cortex carry discriminative information for decoding [3,4,5].

Traditional MI decoding commonly follows a “feature extraction + feature selection + classifier” pipeline. The Common Spatial Pattern (CSP) algorithm is a representative spatial filtering technique that learns linear projections maximizing the variance ratio between two classes, enhancing discriminative oscillatory components in sensorimotor channels [2,6]. To address the frequency-specific nature of MI and improve robustness, Filter Bank CSP (FBCSP) decomposes EEG into multiple sub-bands and applies CSP per band, followed by feature selection and classification [7]. Further variants such as probabilistic CSP (P-CSP) incorporate uncertainty modeling [8], while discriminative FBCSP attempts to optimize class separability more directly [9]. In practice, these CSP-based approaches often rely on additional steps such as selecting informative components using Fisher-type criteria [10] and applying linear classifiers (e.g., LDA) for final decision-making. Although effective, such pipelines are sensitive to preprocessing choices, stationarity assumptions, and inter-session/inter-subject shifts; moreover, they depend heavily on handcrafted features and may not capture complex spatiotemporal patterns in raw EEG [11,12].

Deep learning has increasingly become a dominant approach in EEG/MI decoding because it can learn hierarchical representations directly from raw or minimally preprocessed signals, reducing reliance on handcrafted features [13,14,15,16,17,18]. EEGNet [13] is a compact convolutional neural network (CNN) designed for EEG decoding that combines 1D convolutions (acting as learnable band-pass filters) with depthwise spatial convolutions (acting as data-driven spatial filters), followed by separable convolutions for efficient feature mixing. ShallowConvNet [14] and subsequent refinements [19] mimic elements of classical band-power pipelines by using temporal filtering and nonlinear transformations (e.g., squaring/log) but remain fully end-to-end trainable, yielding strong performance with modest parameter counts. Ingolfsson et al. [20] introduced the Temporal Convolutional Network (TCNet) by incorporating temporal convolution layers into the EEGNet framework. Musallam et al. [21] further refined TCNet by introducing architectural and optimization improvements, enabling high classification accuracy across subjects using a single set of hyperparameters. Motivated by the success of self-attention in sequence modeling [22], Transformer-style components have also been explored in EEG decoding to capture global dependencies beyond local convolutional receptive fields [23,24]. For example, EEG Conformer combines convolutional front-ends with self-attention modules and reports strong performance with additional interpretability via activation/topography visualization [25]. For MI specifically, CTNet has been proposed to jointly learn local spatiotemporal features and global dependencies, showing competitive results in both subject-specific and cross-subject evaluations [26]. ATCNet incorporates attention mechanisms and a sliding-window strategy into TCNet to strengthen temporal modeling and inter-channel dependency learning, achieving competitive results across MI benchmarks [27].

Despite progress, reliable MI classification remains challenging in realistic settings. A key challenge is limited labeled data: collecting high-quality MI EEG requires time-consuming sessions, and subjects often experience fatigue and reduced attention, leading to small numbers of usable trials per class [2,4]. This small-sample regime is further complicated by pronounced nonstationarity across sessions and substantial inter-subject variability caused by differences in anatomy, cognitive strategies, electrode placement, and signal-to-noise ratios [28,29]. As a result, models trained on one subject/session may generalize poorly to another, and deep networks are particularly prone to overfitting when fine-tuned with few target trials. Reducing the dependence on extensive per-subject calibration while maintaining high accuracy has therefore become an important research direction, motivating transfer learning (TL), domain adaptation (DA), and other cross-subject learning strategies [29,30,31,32,33].

Transfer learning aims to leverage knowledge learned from source subjects/sessions to improve performance on a target subject, especially when target samples are scarce. A common and effective strategy in deep learning is “pretraining + fine-tuning”: the network is first pretrained on pooled data from other datasets to learn generalizable representations, then adapted to the target subject via fine-tuning. However, naïve fine-tuning may still overfit to limited target trials and may amplify subject-specific noise, calling for regularization and alignment mechanisms.

Alignment-based methods attempt to reduce distribution mismatch between subjects. Euclidean-space alignment (EA) estimates an alignment transform using second-order statistics so that EEG trials from different subjects are mapped into a more consistent feature space before classification [30]. More generally, Riemannian geometry-based approaches represent EEG trials as symmetric positive definite (SPD) covariance matrices and perform learning or alignment on the SPD manifold, often exhibiting strong robustness to noise and variability [32,34]. In particular, Riemannian Procrustes Analysis (RPA) aligns covariance distributions across subjects/sessions via geometric transforms, enabling effective transfer with reduced calibration [31]. Recent extensions also align subjects in tangent space or combine alignment with end-to-end deep learning, reflecting a broader trend toward geometry-aware transfer [35,36].

Another line of work draws from general domain adaptation in machine learning, where the goal is to learn representations that are both discriminative for labels and invariant across domains [37]. Adversarial DA methods (e.g., DANN) introduce a domain discriminator and a gradient reversal mechanism to encourage domain-invariant features [38]. Moment-matching approaches reduce distribution discrepancies using statistics such as maximum mean discrepancy (MMD) or deep correlation alignment (Deep CORAL) [35]. These generic DA principles have inspired EEG/MI transfer designs and provide a useful conceptual framework for understanding why cross-subject generalization often fails: the model may learn features that separate classes well in the source subjects but still encode subject identity.

Beyond alignment/DA, several strategies specifically target small-sample overfitting during fine-tuning. These include importance reweighting to correct distributional mismatch [39], stochastic regularization objectives in transfer settings [40], freezing or selectively adapting subsets of parameters/layers for MI-specific transfer [41], meta-learning approaches that learn initialization/updates that adapt quickly with few target trials [42], and augmentation-driven auxiliary objectives to improve robustness [43]. Nevertheless, in practical MI decoding, there remains a need for simple, architecture-agnostic mechanisms that can stabilize fine-tuning and preserve discriminative representations under limited target data.

In rehabilitation-oriented MI-BCI training, models must adapt from brief calibration and remain stable across repeated sessions to support reliable feedback, motivating transfer learning strategies that are robust in the low-calibration regime. In this work, we propose a transfer learning strategy named Maximizing Single-Feature Separability (MSFS) for subject-dependent MI EEG classification. Our hypothesis is that, in a trained network, the feature layer (i.e., the representation immediately before the final classifier) contains a subset of individual feature dimensions that already exhibit meaningful class separability. During fine-tuning with scarce target trials, however, non-discriminative features may spuriously correlate with each other and begin to contribute to the classifier, which might be an indicator of overfitting. To counteract this drift, MSFS introduces an auxiliary objective that explicitly encourages each individual feature dimension to remain discriminative with respect to the classes.

Specifically, we use the silhouette coefficient—a classical cluster separability measure [44]—to quantify how well samples of different classes separate along each feature. During fine-tuning, we compute feature-wise target positions that maximize silhouette separability within each batch and impose a regression-style constraint that pulls features toward these optimal positions. Because the target positions depend on the batch composition, shuffling data each epoch naturally yields a stochastic auxiliary objective, acting as regularization and reducing the risk of memorizing idiosyncratic target–subject noise. Importantly, MSFS is model-agnostic and can be integrated into representative MI networks without modifying their main architectures, which facilitates its use in practical MI-BCI pipelines where calibration time and robustness are key considerations.

The main contributions of this work are summarized as follows:

1. Intra-dataset transfer learning for low-calibration MI-BCI: We leverage data from other subjects within the same dataset to improve target–subject classification without introducing external datasets, thereby mitigating the few-sample calibration problem commonly encountered in practical MI-BCI use.
2. Feature-level discriminability enhancement: We employ the Silhouette coefficient to quantify class separability of each learned feature dimension and introduce an auxiliary objective that preserves single-feature discriminability during fine-tuning, improving accuracy and robustness under limited target trials.
3. Stochastic auxiliary regularization to reduce overfitting: By computing batch-dependent optimal feature targets, random shuffling induces a stochastic auxiliary objective across iterations, regularizing adaptation and reducing sensitivity to idiosyncratic training-set noise, which is important for stable performance across sessions.

The remainder of this paper is organized as follows. Section 2 provides a detailed description of the proposed MSFS architecture. Section 3 presents the experimental results, including performance comparison against models from the literature and an ablation analysis to evaluate the contribution of each proposed enhancement. Section 4 analyzes the experimental results. Finally, Section 5 concludes the paper and discusses potential directions for future work.

## 2. Methods

### 2.1. Problem Definition and Notation

We consider an EEG MI classification task with *K* classes. For each trial, the input EEG segment is denoted as

(1) $$x\in R^{C\times T},$$

where *C* is the number of channels and *T* is the number of time points. The corresponding label is y∈{1,…,K}.

A neural network is decomposed into a feature extractor $f_{\theta }\left(\cdot \right)$ and a classifier head $g_{\phi }\left(\cdot \right)$. For a batch ${\left\{\left(x_{i},y_{i}\right)\right\}}_{i=1}^{N}$, the feature vector and logits are

(2) $$z_{i}=f_{\theta }\left(x_{i}\right)\in R^{F},\;\ell_{i}=g_{\phi }\left(z_{i}\right)\in R^{K},$$

and the predicted class probabilities are $p_{i}=\mathrm{softmax}\left(\ell_{i}\right)$.

### 2.2. Datasets and Input Construction

We evaluate MSFS on the BCI Competition IV datasets 2a and 2b [24].

- BCI IV-2a contains EEG from 22 channels and nine subjects. Each subject has two sessions (training and testing), each with 288 trials. Signals are sampled at 250 Hz and processed with a 0.5–100 Hz band-pass filter. The MI task includes four classes (left hand, right hand, foot, tongue).
- BCI IV-2b contains EEG from three channels and nine subjects. Each subject has five sessions. Sessions 1–2 are recorded with feedback, and sessions 3–5 are recorded without feedback. We use sessions 1–3 for training and sessions 4–5 for testing. Signals are sampled at 250 Hz and processed with a 0.5–100 Hz band-pass filter. The task includes two classes (left vs. right hand).

We perform no additional preprocessing and no data augmentation to keep the pipeline end-to-end and consistent with prior deep MI decoding practices (e.g., ATCNet). Each trial is obtained by directly cropping the raw EEG around the cue onset, using a 4.5 s segment including 0.5 s before and 4 s after the cue, resulting in T=1125 samples per trial at 250 Hz. The cropped trials are fed into networks as input tensors.

### 2.3. Base Networks

We adopt three representative deep MI classifiers: EEGNet [13], ShallowConvNet [14], and ATCNet [27]. For EEGNet and ShallowConvNet, we follow the original architectures and hyperparameters reported in their respective papers. For ATCNet, we also follow the original design and additionally clarify the decision strategy used in this work: ATCNet has a sliding-window module and multiple parallel branches. Let $\ell_{i}^{\left(b\right)}\in R^{K}$ denote the logit of branch *b*. The final output logit is computed by averaging branch logits:

(3) $$\ell_{i}=\frac{1}{B}\sum_{b=1}^{B}\ell_{i}^{\left(b\right)}.$$

In EEGNet and ShallowConvNet, the feature layer refers to the layer immediately preceding the final dense layer that outputs logits (i.e., the input of the final dense layer). In ATCNet, which contains multiple branches, each branch produces a logit; the feature layer refers to the layer immediately preceding the final dense layer within each branch.

### 2.4. Transfer Learning Protocol and Hyperparameter Selection

**Figure 1:**
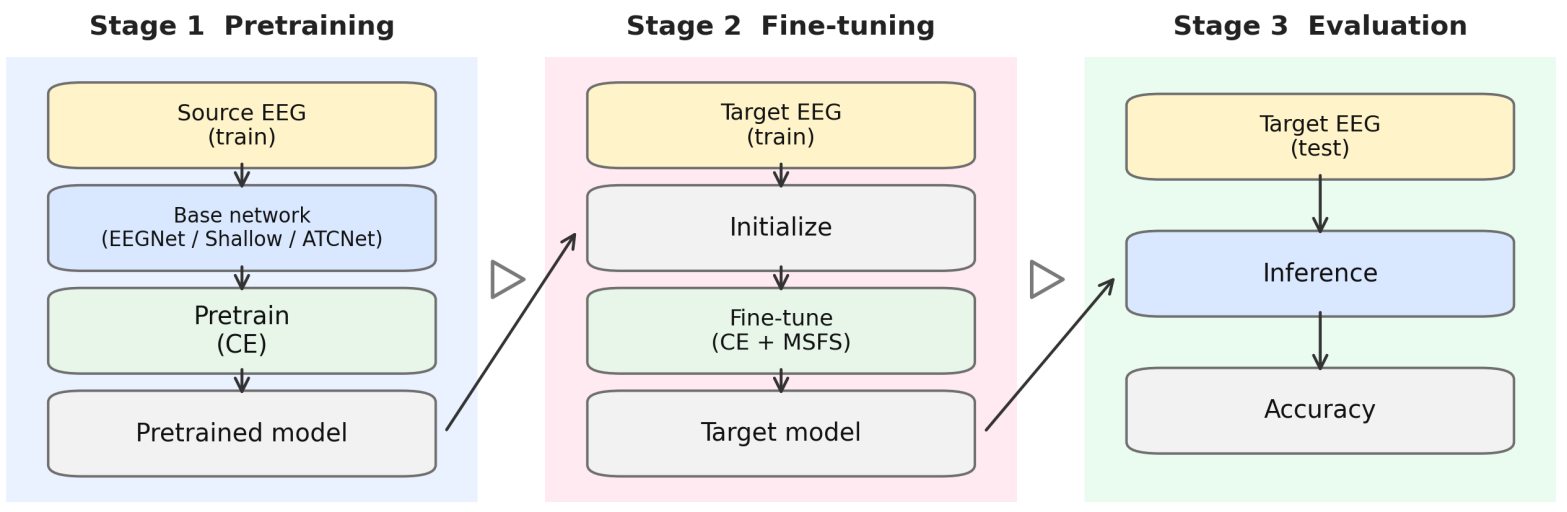
Overall pipeline of intra-dataset transfer learning with MSFS.

We use the following transfer setting: for each target subject, we pretrain the model on all other subjects (source subjects) and then fine-tune on the target subject. The overall pipeline is shown in Figure 1.

Pretraining stage: The model is trained on pooled data from all source subjects to learn a subject-independent initialization. Only cross-entropy (CE) loss is used in this stage.

Fine-tuning stage: The pretrained model is adapted using the target subject’s training data. In fine-tuning, MSFS is activated to regularize feature learning (Section 2.5).

All MSFS-related hyperparameters are selected using a leave-one-subject-out protocol on the training set. For each target subject, we randomly split the target subject’s available training trials within each class into two equal parts: one for fine-tuning training and the other for validation. Hyperparameters are chosen to maximize the mean validation accuracy averaged across all target subjects. The final reported test results are then obtained by training with the selected hyperparameters and evaluating on the predefined test set.

### 2.5. MSFS: Maximizing Single-Feature Separability During Fine-Tuning

#### 2.5.1. Motivation and Overall Objective

During fine-tuning with limited target data, deep models may over-adapt and begin exploiting non-discriminative features. MSFS introduces a feature-level auxiliary objective that encourages every single feature to maintain class separability. Importantly, we do not apply MSFS from the beginning of fine-tuning because adding stochastic regularization too early can destabilize optimization; instead, MSFS is activated only after the model has reached a sufficiently low classification loss.

#### 2.5.2. Silhouette-Based Target Positions and Feature Regression Loss

The standard cross-entropy loss is

(4) $$L_{\mathrm{CE}}=-\frac{1}{N}\sum_{i=1}^{N}\log p_{i,y_{i}}.$$

For each batch, MSFS computes a batch-dependent target position matrix $t\in R^{N\times F}$ for the feature matrix $Z\in R^{N\times F}$, such that single-feature class separability (measured by the silhouette coefficient) is maximized. We use the absolute distance for one-dimensional feature values:

(5) $$d_{f}\left(x_{i},x_{k}\right)=\left|z_{i,f}-z_{k,f}\right|.$$

Following the silhouette definition [44], for each sample *i*, let $a_{i}$ be the mean intra-class distance and $b_{i}$ be the minimum mean inter-class distance, defined respectively as

(6) $$a_{i}=\frac{1}{|C_{y_{i}}|-1}\sum_{x_{k}\in C_{y_{i}},\;k\neq i}d_{f}\left(x_{i},x_{k}\right),$$

(7) $$b_{i}=\min_{q\neq y_{i}}\frac{1}{|C_{q}|}\sum_{x_{k}\in C_{q}}d_{f}\left(x_{i},x_{k}\right),$$

where $C_{q}$ is the set of samples in class *q*, the silhouette score is

(8) $$s_{i}=\frac{b_{i}-a_{i}}{\max(a_{i},b_{i})}.$$

MSFS searches, for each (i,f), a candidate value among existing batch feature values ${\left\{z_{j,f}\right\}}_{j=1}^{N}$ and selects the candidate that maximizes the silhouette score under the condition that other samples are fixed. The selected candidate value becomes the target $t_{i,f}$ for the feature $z_{i,f}$.

**Figure 2:**
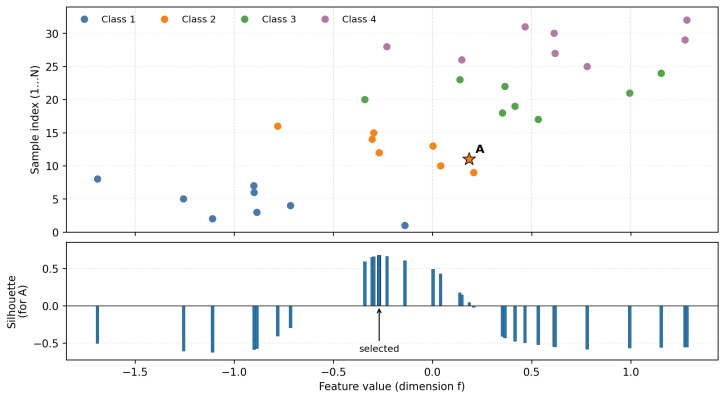
Example of target position selection in MSFS.

Figure 2 shows an example of target position selection in MSFS. The upper and lower panels share the same horizontal axis, which represents the feature value along feature dimension *f*. The upper panel is a scatter plot showing the feature values of N = 32 samples in a mini-batch, where we assume eight samples per class. The vertical axis indicates the sample indices. We seek a target position for sample *A*, which belongs to Class 2 and is highlighted by an orange star. Candidate target positions are given by the feature values of all samples in the current mini-batch. The lower panel presents a histogram of the silhouette score obtained when relocating *A* to each candidate position. As shown, candidates far from the Class 2 cluster yield low silhouette values, sometimes even negative, whereas candidates closer to the cluster center produce higher silhouette values. We select the candidate with the maximum silhouette score as the target position for *A*. The target positions of the remaining samples in the mini-batch are determined in the same manner.

Given the target matrix *t*, MSFS defines a mean squared error objective:

(9) $$L_{\mathrm{pos}}=\frac{1}{NF}\sum_{i=1}^{N}\sum_{f=1}^{F}{\left(z_{i,f}-t_{i,f}\right)}^{2}.$$

MSFS is activated only when the batch cross-entropy loss is below a threshold ${\mathrm{Loss}}_{\min}$, yielding a hard-gated objective:

(10) $$L=L_{\mathrm{CE}}+\omega \cdot 1\;\left(L_{\mathrm{CE}}<{\mathrm{Loss}}_{\min}\right)\cdot L_{\mathrm{pos}},$$

where ω controls the contribution of the MSFS loss.

When a batch contains any class with ≤1 samples, the silhouette computation for that class becomes ill-defined. In such cases, we skip MSFS for that batch and optimize only $L_{\mathrm{CE}}$. This situation is rare in our experiments and has negligible impact on results.

#### 2.5.3. Vectorized Computation and Complexity

The determination of target position is implemented in a fully vectorized manner across samples and features. In brief, for each feature dimension *f*, we compute the pairwise distance tensor

(11) $$D\left[f,j,k\right]=|z_{j,f}-z_{k,f}|,$$

then aggregate within-class and between-class mean distances to compute silhouettes for all candidate replacements simultaneously, and finally take an argmax over candidate indices *j* to obtain $t_{i,f}=z_{j^{*},f}$. This results in an approximate per-batch time complexity of

(12) $$O(FN^{2}),$$

which is efficient in practice under GPU vectorization with typical batch sizes.

### 2.6. Algorithm Summary

Algorithm 1 summarizes the complete training procedure (pretraining + fine-tuning with gated MSFS). For each mini-batch during fine-tuning, a forward pass was performed to obtain both the *logit* and the intermediate *feature* representations. The function CE_Loss computes the cross-entropy loss between the predicted logit and the ground-truth label. If the obtained loss value was smaller than a predefined minimum threshold $Loss_{min}$, the optimization process proceeded to the MSFS computation stage. The function Opt_Pos_Sil was employed to determine the optimal target feature positions $t_{pos}$ for all samples within the batch. The mean squared error between the network features and $t_{pos}$, denoted as $loss_{pos}$, was then calculated by the function MSE_Loss. Finally, model parameters were updated by backpropagation with respect to loss.
**Algorithm 1** Training with MSFS**Input:** number of subjects *n*, training data of all subjects *D*[1:*n*], training label of all subjects *L*[1:*n*], number of pre-training epochs $e_{p}$, number of fine-tune epochs $e_{f}$, batch size *b*, minimal loss $Loss_{min}$, loss weight *w***Output:** trained model of all subjects *M*1: M← void list2: **for** sub=1 to *n* **do**3:       $M_{s}\leftarrow$ initial model4:       $D_{s}$, $L_{s}$← training data and label of all subjects except sub from *D* and *L*5:       Pre-train $M_{s}$ with $D_{s}$ and $L_{s}$ for $e_{p}$ epochs6:       $D_{s}$, $L_{s}$← training data and label of subject sub from *D* and *L*7:       Loader← DataLoader($D_{s}$, $L_{s}$, batch_size = *b*, shuffle = True)8:       **for** e=1 to $e_{f}$ **do**9:       **for** data, label in Loader **do**10:        logit, feature←$M_{s}$.forward(data)11:        loss← CE_Loss(logit, label)12:        **if** loss < $Loss_{min}$ **then**13:          $t_{pos}\leftarrow$ Opt_Pos_Sil(feature, label)14:          $loss_{pos}\leftarrow$ MSE_Loss(feature, $t_{pos}$)15:          loss←loss + $loss_{pos}$*w16:        **end if**17:        Back propagate loss18:       **end for**19:       **end for**20:       Add $M_{s}$ to *M*21: **end for**22: **return** *M*

To promote training stability and prevent the network from converging toward a fixed feature configuration, the training data were randomly shuffled at the beginning of each epoch. This randomization ensures that the samples in each batch used by the Opt_Pos_Sil function vary across iterations, introducing stochasticity into the computed optimal positions and enhancing generalization.

Algorithm 2 provides the vectorized computation of the silhouette-based target positions (function Opt_Pos_Sil) used in MSFS.
**Algorithm 2** Function Opt_Pos_Sil**Input:** Feature matrix $X\in R^{N\times F}$, label vector $y\in {\left\{1,\ldots ,K\right\}}^{N}$**Output:** Optimal position matrix $\mathrm{Opt}\in R^{N\times F}$1: Compute pairwise distances $D\left[f,j,k\right]=\;|X_{j,f}-X_{k,f}|$2: For each class *c*, compute mean distances $b_{c}\left[f,j\right]=\frac{1}{|Class_{c}|}\sum_{k\in Class_{c}}D\left[f,j,k\right]$3: For each sample *i*, derive within-class means $a\left[i,f,j\right]=\frac{1}{|Class_{y_{i}}|-1}\sum_{k\in Class_{y_{i}},\;k\neq i}D\left[f,j,k\right]$4: For each sample *i*, obtain between-cluster distances $b\left[i,f,j\right]=\min_{c\neq y_{i}}b_{c}\left[f,j\right]$5: Compute silhouette values $s\left[i,f,j\right]=\frac{b[i,f,j]-a[i,f,j]}{\max(a[i,f,j],b[i,f,j])}$6: For each (i,f), find $j^{*}=\arg\max_{j}s\left[i,f,j\right]$7: Assign optimal positions ${\mathrm{Opt}}_{i,f}=X_{j^{*},f}$8: **return** Opt

## 3. Experiments

### 3.1. Experimental Settings

We conduct experiments on the BCI Competition IV datasets 2a and 2b. For BCI IV-2a, we follow the official split by using the training session for model training and the testing session for evaluation. For BCI IV-2b, we adopt a commonly used session split: sessions 1–3 are used for training and sessions 4–5 are used for testing. In the evaluation of transfer learning, each subject is treated as the target subject, while the remaining subjects constitute the source domain. The model is first pretrained on the pooled source-subject data and then fine-tuned on the target subject’s training set. MSFS is applied only during the fine-tuning stage.

All backbones (EEGNet, ShallowConvNet, and ATCNet) are optimized using Adam (PyTorch 2.0, Python 3.9) with a fixed learning rate of 0.001 and weight decay of 0.009. The batch size is set to 64. To account for randomness in optimization, all results are reported as the average of three independent runs.

Hyperparameters are selected using training data only. Specifically, for each target subject, the target training trials are randomly split in a class-balanced manner into two equal parts: one part is used for fine-tuning training and the other for validation. Hyperparameters are determined by maximizing the mean validation accuracy averaged across target subjects. The final evaluation is performed once on the target test set using the selected hyperparameters. Performance is measured using classification accuracy.

Table 1 summarizes the hyperparameters used in the final experiments for each dataset–backbone combination, including the pretraining epochs ($e_{p}$), fine-tuning epochs ($e_{f}$), MSFS weight (ω), and the gating threshold ($Loss_{min}$).

### 3.2. MSFS on Two Datasets and Three Backbones

In this section, we evaluate the effectiveness and generality of MSFS under the transfer learning setting on two MI datasets (BCI IV-2a and IV-2b) and three representative deep EEG classifiers (EEGNet, ShallowConvNet, and ATCNet). We compare the following training strategies:

1. Original: the model is trained only on the target subject’s training set, without using data from other subjects.
2. Standard transfer learning (TL, CE only): the model is pretrained on the pooled source subjects and then fine-tuned on the target subject using cross-entropy loss only.
3. TL + MSFS (ours): the same pretraining and fine-tuning procedure is used, while MSFS is additionally applied during fine-tuning.

All results are reported as mean accuracy and subject-level Wilcoxon *p*-values over three independent runs. Hyperparameters for each dataset–backbone pair are fixed as summarized in Table 1. The main results on BCI IV-2a and BCI IV-2b are presented in Table 2 and Table 3, respectively.

Overall, standard transfer learning consistently outperforms subject-dependent training, indicating the benefit of leveraging source-subject data for initialization. More importantly, TL + MSFS further improves performance over CE-only fine-tuning across different backbones on both datasets, demonstrating that MSFS provides a backbone-agnostic regularization effect that is beneficial for target–subject adaptation.

### 3.3. Comparison to Literature Models

To further position MSFS against representative transfer-learning and domain-adaptation approaches in the literature, we compare our method with the following baselines: EEGNet (without TL), Transfer Learning (EEGNet with Standard TL), EA (Euclidean Alignment) [30], Adaptive TL [41], MixDual-Tuning [43], Meta-learning [42], and DIW (Dynamic Importance Weighting) [39]. For a fair comparison, all methods use EEGNet as the backbone and are evaluated under the same transfer learning protocol on BCI IV-2a and BCI IV-2b. We select EEGNet for literature baselines because many prior TL or DA methods were originally reported with EEGNet, enabling a faithful reproduction under a unified pipeline.

All results reported in this section are obtained from our own reproductions under a unified training and evaluation pipeline (Section 3.1). For each target subject, the model is pretrained on source subjects and fine-tuned on the target subject. Hyperparameters are selected according to the original paper.

Table 4 and Table 5 report per-subject accuracies on IV-2a and IV-2b, respectively. The experimental results demonstrate that applying transfer learning notably improved classification performance compared to the original EEGNet, indicating that this framework can effectively increase classification accuracy across the dataset without introducing external data. The five methods—EA, DIW, Meta-Learning, Adaptive Transfer Learning, and MixDual-Tuning—when combined with standard transfer learning, did not achieve significant performance gains over standard transfer learning. Among them, only Adaptive Transfer Learning slightly outperformed the standard transfer learning baseline, suggesting limited improvement under the evaluated settings. In contrast, the proposed MSFS method achieved a substantial accuracy increase, raising the average classification accuracy from 0.7616 to 0.8184 for BCI IV-2a and moderate improvement for 2b, validating the effectiveness of the MSFS framework.

### 3.4. Ablation Studies

To validate the key design choices of MSFS, we conduct two ablation studies targeting (i) the effectiveness of the silhouette-based target position optimization and (ii) the necessity of the gated activation strategy.

#### 3.4.1. Rand_Pos: Effect of Silhouette-Based Target Positions

In MSFS, the target position matrix t is determined by maximizing silhouette scores within each batch. To assess whether the improvement is indeed brought by the silhouette-driven optimization (rather than simply adding an auxiliary regression loss), we replace the optimized target positions with random target positions, referred to as Rand_Pos, which is presented in Algorithm 3. Rand_Pos uses the same loss form as MSFS, but removes the separability criterion.
**Algorithm 3** Function Rand_Pos**Input:** Feature matrix $X\in R^{N\times F}$**Output:** Random position matrix $\mathrm{Opt}\in R^{N\times F}$1: **for** each feature f=1 to *F* **do**2:    Compute $L_{f}=\min_{1\leq j\leq N}X_{j,f}$    ▹ Minimum value in feature *f*3:    Compute $U_{f}=\max_{1\leq j\leq N}X_{j,f}$     ▹ Maximum value in feature *f*4:    **for** each sample i=1 to *N* **do**5:    Draw ${\mathrm{Opt}}_{i,f}$ uniformly from $\left[L_{f},\;U_{f}\right]$6:    **end for** 7:**end for** 8:**return** Opt

#### 3.4.2. No-Gating: Effect of the Gated Activation

MSFS is designed to be activated only when the batch loss becomes sufficiently small. This gating is introduced to avoid injecting stochastic regularization at the beginning of fine-tuning, which may impair optimization stability. In the No-gating variant, we apply MSFS throughout fine-tuning (i.e., the MSFS term is always active), while keeping all other settings identical.

#### 3.4.3. Results and Analysis

Table 6 summarizes the ablation results on both datasets (BCI IV-2a and IV-2b) and all three backbones. Overall, MSFS achieves the best performance across most dataset–backbone combinations, indicating that both components are important. Rand_Pos consistently underperforms MSFS, suggesting that maximizing silhouette score provides meaningful target positions that better preserve class separability at the feature level. No-gating tends to degrade performance compared with MSFS, supporting the hypothesis that enabling MSFS too early can disturb optimization and that the proposed gating strategy improves stability during fine-tuning.

It is also observed that even with random feature targets, the accuracy remained higher than that of standard transfer learning, suggesting that introducing stochastic guidance at the feature level can itself provide a beneficial regularization effect. This form of randomness is preserved in the proposed MSFS framework through the shuffling of training samples in each epoch of Algorithm 1, ensuring that every batch yields distinct feature–label pairings and thereby generating naturally varying $t_{pos}$ values across iterations.

### 3.5. Few-Shot Target Data Study

In practical MI-BCI scenarios, only a limited amount of labeled data may be available for a new user. To evaluate the robustness of MSFS under scarce target supervision, we conduct a few-shot target-data study where the fine-tuning set of the target subject is subsampled at different labeled-data budgets.

For each target subject, we perform stratified sampling on the target training set by selecting 10%, 25%, 50%, and 100% of trials from each class, ensuring class balance at every budget level. For each budget, we compare:

1. TL (CE only): pretrain on source subjects and fine-tune on the subsampled target data using cross-entropy loss only.
2. TL + MSFS (ours): the same procedure, with MSFS additionally applied during fine-tuning.

All other settings follow Section 3.1. Table 7 summarizes the results on BCI IV-2a and IV-2b using the EEGNet backbone. In general, performance improves monotonically with more labeled target data for both methods. MSFS still provides large relative gains in the low-data regime (10% and 25%).

### 3.6. Efficiency

MSFS introduces an additional per-mini-batch computation to determine silhouette-optimized target positions and compute the auxiliary regression loss. To examine the practical overhead, we report the average training time per epoch for standard transfer learning (TL, CE-only) and TL with MSFS (TL+MSFS) across both datasets and all three backbones.

As summarized in Table 8, TL + MSFS incurs only a modest increase in per-epoch training time compared with TL. This is because the core routine of MSFS is implemented in a fully vectorized manner and is executed efficiently on the GPU. Overall, the results indicate that MSFS improves adaptation performance with moderate additional computational cost, making it easy to incorporate into real training pipelines. Timing was measured on an NVIDIA GeForce RTX 4090 GPU (batch size = 64).

Despite the modest overhead observed in our current setting, the exact MSFS computation scales with the number of feature dimensions and the batch size due to the pairwise distance terms in the 1D silhouette (approximately $O(FN^{2})$). In practice, several approximations can substantially reduce this cost without changing the overall training pipeline. First, the silhouette target position for each feature can be estimated using candidate subsampling, i.e., evaluating the objective on a small set of values per class (randomly sampled from the batch), rather than enumerating all possible targets. Second, MSFS can be applied only to the top-K most informative feature dimensions, identified by separability statistics. These approximations provide a natural path to extend MSFS to larger batches and higher-dimensional representations while retaining its core objective of regularizing fine-tuning.

## 4. Discussion

This work proposed MSFS, a simple and architecture-agnostic feature-level regularization strategy for transfer learning in MI EEG classification. Across two public benchmarks (BCI IV-2a/2b) and three representative backbones (EEGNet, ShallowConvNet, and ATCNet), MSFS consistently improved the standard “pretrain + fine-tune” baseline, indicating that enforcing single-feature discriminability can stabilize target–subject adaptation under limited labeled data. On BCI IV-2a, the gain is especially pronounced for EEGNet (from 0.7616 to 0.8184 in mean accuracy), while improvements remain positive for ShallowConvNet and ATCNet. On BCI IV-2b, the absolute gains are smaller but still consistent across backbones, suggesting that MSFS remains beneficial even when baseline performance is already high. Such changes can still be meaningful in MI-BCI: small average improvements that are consistent across subjects may translate into noticeably reduced calibration effort and more reliable control for individual users. These observations support the central premise that fine-tuning on scarce target trials may introduce spurious feature co-adaptation, and that preserving per-dimension separability provides an effective regularization.

The ablation studies provide evidence that both components of MSFS contribute to performance. First, replacing silhouette-optimized targets with random targets (Rand_Pos) consistently reduces accuracy compared with MSFS on most dataset–backbone combinations, implying that the silhouette criterion is not merely injecting noise but is producing meaningful target directions that better preserve class structure at the feature level. Second, removing the loss-based activation gate (No-gating) typically degrades performance relative to MSFS, supporting the hypothesis that applying the auxiliary objective too early can interfere with optimization before the classifier becomes sufficiently discriminative. Notably, Rand_Pos can still remain competitive with (and sometimes close to) MSFS, which suggests that even stochastic feature-level guidance may yield a mild regularization benefit; MSFS retains this beneficial stochasticity while further steering it toward increased separability through silhouette maximization.

Although MSFS introduces additional computations for batch-wise target position estimation, the time-per-epoch measurements indicate only a modest overhead across both datasets and all three backbones. This is largely due to the fully vectorized implementation of the silhouette-based target computation, which is amenable to GPU acceleration. Therefore, MSFS offers a favorable trade-off: measurable accuracy improvements with limited additional training time, making it easy to incorporate into existing transfer learning pipelines.

Although the silhouette coefficient is typically used as a global clustering measure in multi-dimensional spaces, we use it as a per-feature (1D) discriminability proxy to regularize target-domain fine-tuning. From a representation learning perspective, this auxiliary objective can be viewed as a lightweight constraint that mitigates representation drift under scarce target supervision by encouraging a favorable trade-off between within-class compactness and between-class separation along individual feature dimensions. MSFS is not intended to replace joint-feature decision making: the standard cross-entropy loss still optimizes the classifier in the full feature space and captures multi-feature interactions, while MSFS provides a complementary regularization that helps preserve informative feature dimensions. MSFS may be less effective when discriminative information of the original model primarily arises from strong feature interactions.

In terms of online or adaptive MI-BCI deployment, MSFS in its current form is a supervised fine-tuning regularizer and thus relies on access to target labels. In practice, labels may be delayed or noisy. A straightforward way to accommodate delayed labels is to apply MSFS in buffered updates, where recent trials are accumulated, and the model is updated intermittently once labels become available. For noisy labels, MSFS could be applied by weighting the auxiliary objective using soft labels or uncertainty estimates (e.g., posterior variance from Bayesian approximations). Exploring these label-efficient and noise-robust extensions constitutes an important direction for future work.

Overall, the results suggest that preserving single-feature discriminability is a simple yet effective principle for stabilizing deep fine-tuning under target data scarcity. MSFS provides a lightweight plug-in regularization that improves transfer learning for MI EEG classification across multiple backbones and datasets.

## 5. Conclusions

This paper presented MSFS, a lightweight and architecture-agnostic regularization strategy for transfer learning in motor imagery EEG classification. MSFS augments standard TL pipelines by encouraging feature-level class separability during target–subject adaptation through a silhouette-driven target-position mechanism with a loss-based activation gate.

Extensive experiments on BCI Competition IV-2a and IV-2b with three representative backbones (EEGNet, ShallowConvNet, and ATCNet) demonstrate that MSFS consistently improves CE-only fine-tuning. Comparisons with reproduced literature baselines further confirm the competitiveness of MSFS under a unified EEGNet-based evaluation. Ablation studies validate the contributions of the silhouette-based target optimization and the gated activation, while the few-shot study indicates that MSFS is beneficial when only limited labeled target data are available. Finally, efficiency results show that the proposed vectorized implementation introduces only a modest computational overhead.

Overall, MSFS offers a plug-in module that strengthens target–subject adaptation with minimal additional cost, which may support more usable MI-BCI rehabilitation workflows by shortening calibration and improving reliability of feedback-driven training (e.g., cue-based neurofeedback or device-assisted therapy). The present study is scoped to within-dataset transfer, which limits the conclusions we can draw about MSFS under more challenging distribution shifts, such as cross-session variability, cross-hardware shifts, and cross-dataset transfer, where additional non-stationarities may be more pronounced. Future work will explore combining MSFS with alignment or self-supervised pretraining techniques and extending the evaluation to more challenging adaptation scenarios (e.g., cross-session and cross-dataset transfer).

## Figures and Tables

**Table 1:** Hyperparameters used in final experiments. $e_{p}$ and $e_{f}$ denote the number of pretraining and fine-tuning epochs, respectively. ω is the MSFS loss weight, and ${\mathrm{Loss}}_{\min}$ is the gating threshold.

Dataset	Backbone	$e_{p}$	$e_{f}$	ω	${\mathrm{Loss}}_{\min}$
BCI IV-2a	EEGNet	500	200	1	0.2
BCI IV-2a	ShallowConvNet	500	100	0.5	0.1
BCI IV-2a	ATCNet	700	200	0.5	0.2
BCI IV-2b	EEGNet	500	100	0.5	0.2
BCI IV-2b	ShallowConvNet	500	50	1	0.2
BCI IV-2b	ATCNet	700	200	1	0.1

**Table 2:** Main results on BCI IV-2a. We report mean accuracy and *p*-values (in the bracket) over three runs, *p*-values are from subject-level Wilcoxon signed-rank tests comparing MSFS vs. baseline on per-subject scores averaged over 3 runs (two-sided). * indicates *p* < 0.05, ** indicates *p* < 0.01. Rows correspond to backbones and columns correspond to training strategies. Original: subject-dependent training using only target–subject training data. TL: pretrain on source subjects and fine-tune on the target subject with CE loss only. TL + MSFS: TL with MSFS applied during fine-tuning.

Backbone	Original	TL (CE Only)	TL + MSFS
EEGNet	0.7231 (** 0.0039)	0.7616 (** 0.0039)	0.8184
ShallowConvNet	0.6745 (** 0.0039)	0.7591 (* 0.0195)	0.7698
ATCNet	0.7939 (** 0.0039)	0.8278 (** 0.0078)	0.8502

**Table 3:** Main results on BCI IV-2b. The evaluation protocol and notations follow Table 2.

Backbone	Original	TL (CE Only)	TL + MSFS
EEGNet	0.8555 (0.0742)	0.8605 (0.0929)	0.8667
ShallowConvNet	0.8352 (** 0.0039)	0.8672 (0.7794)	0.8680
ATCNet	0.8525 (0.2031)	0.8537 (0.6744)	0.8583

**Table 4:** Per-subject accuracy on BCI IV-2a using EEGNet backbone (mean over three runs). Ax refers to the x th subject in BCI IV-2a. All the literature baselines are reproduced in our unified pipeline. The highest accuracy for each subject and the average are highlighted in bold.

Method	A1	A2	A3	A4	A5	A6	A7	A8	A9	Avg
EEGNet (Original)	0.7997	0.5878	0.8642	0.6365	0.7149	0.5615	0.7517	0.7837	0.8083	0.7231
TL (CE only)	0.8426	0.6563	0.9120	0.7153	0.6192	0.6238	0.8924	0.8137	0.7789	0.7616
TL + MSFS (ours)	**0.8799**	**0.7222**	**0.9375**	**0.8153**	0.7500	**0.6895**	**0.9090**	**0.8424**	**0.8201**	**0.8184**
EA [30]	0.8657	0.6736	0.8958	0.7222	0.6644	0.6157	0.8877	0.7627	0.7176	0.7562
Adaptive TL [41]	0.8264	0.6447	0.9016	0.7234	**0.7650**	0.6412	0.8275	0.7743	0.8067	0.7679
MixDual-Tuning [43]	0.8021	0.6076	0.8924	0.7419	0.7199	0.6123	0.8854	0.7870	0.7361	0.7539
Meta-learning [42]	0.8021	0.5775	0.8681	0.6736	0.7350	0.6076	0.8843	0.7766	0.7303	0.7395
DIW [39]	0.8137	0.6262	0.9016	0.7002	0.7095	0.6528	0.8113	0.8229	0.7465	0.7539

**Table 5:** Per-subject accuracy on BCI IV-2b using EEGNet backbone (mean over three runs). Bx refers to the x th subject in BCI IV-2b. All the literature baselines are reproduced in our unified pipeline. The highest accuracy for each subject and the average are highlighted in bold.

Method	B1	B2	B3	B4	B5	B6	B7	B8	B9	Avg
EEGNet (Original)	0.7052	0.7107	0.8427	0.9667	0.9594	0.8281	0.9219	0.9167	0.8479	0.8555
TL (CE only)	0.7427	0.6881	0.8354	0.9615	0.9656	0.8490	0.9156	0.9313	0.8552	0.8605
TL + MSFS (ours)	0.7375	0.6929	**0.8542**	**0.9698**	0.9625	0.8698	**0.9240**	0.9333	**0.8563**	**0.8667**
EA [30]	0.7208	**0.7143**	0.8406	0.9604	0.9594	**0.8729**	0.9198	0.9385	0.8344	0.8623
Adaptive TL [41]	0.7490	0.6916	0.8406	0.9646	0.9646	0.8375	**0.9240**	0.9375	0.8677	0.8641
MixDual-Tuning [43]	0.7125	0.6940	0.8448	0.9385	0.9542	0.8510	0.9177	0.9417	0.8479	0.8558
Meta-learning [42]	**0.7750**	0.6976	0.8281	0.9583	**0.9688**	0.8490	0.9042	0.9146	0.8271	0.8581
DIW [39]	0.7135	0.7071	0.8375	0.9635	0.9333	**0.8729**	0.9208	**0.9427**	0.8073	0.8554

**Table 6:** Ablation results. We report mean accuracy and *p*-values (in the bracket) over three runs, *p*-values are from subject-level Wilcoxon signed-rank tests comparing MSFS vs. baseline on per-subject scores averaged over 3 runs (two-sided). * indicates *p* < 0.05, ** indicates *p* < 0.01. Rand_Pos replaces the silhouette-optimized target positions with random positions. No-gating applies MSFS throughout fine-tuning without the loss-based activation gate.

Dataset	Backbone	MSFS (Ours)	Rand_Pos	No-Gating
BCI IV-2a	EEGNet	0.8184	0.8030 (* 0.0117)	0.8058 (0.0976)
BCI IV-2a	ShallowConvNet	0.7698	0.7582 (* 0.0078)	0.7639 (0.4257)
BCI IV-2a	ATCNet	0.8502	0.8306 (** 0.0039)	0.8451 (0.3007)
BCI IV-2b	EEGNet	0.8667	0.8619 (0.3593)	0.8523 (0.0546)
BCI IV-2b	ShallowConvNet	0.8680	0.8688 (0.7343)	0.8673 (0.6523)
BCI IV-2b	ATCNet	0.8583	0.8566 (0.6523)	0.8552 (0.3593)

**Table 7:** Few-shot target-data study using EEGNet backbone. We report mean accuracy and *p*-values (in the bracket) over three runs, *p*-values are from subject-level Wilcoxon signed-rank tests comparing MSFS vs. baseline on per-subject scores averaged over 3 runs (two-sided). ** indicates *p* < 0.01. For each target subject, the fine-tuning set is subsampled with class-balanced (stratified) selection at different budgets. TL: CE-only fine-tuning. TL + MSFS: fine-tuning with MSFS.

Dataset	Method	10%	25%	50%	100%
BCI IV-2a	TL (CE only)	0.6525 (0.3007)	0.6974 (0.0976)	0.7220 (** 0.0039)	0.7616 (** 0.0039)
BCI IV-2a	TL + MSFS (ours)	0.6664	0.7182	0.7744	0.8184
BCI IV-2b	TL (CE only)	0.8487 (0.7343)	0.8550 (0.7343)	0.8562 (0.6523)	0.8605 (0.0929)
BCI IV-2b	TL + MSFS (ours)	0.8505	0.8535	0.8611	0.8666

**Table 8:** Efficiency comparison in terms of average training time per epoch (seconds). TL: CE-only transfer learning. TL + MSFS: transfer learning with MSFS during fine-tuning.

Dataset	Backbone	TL (s/epoch)	TL + MSFS (s/epoch)
BCI IV-2a	EEGNet	$2.347\times 10^{-2}$	$2.714\times 10^{-2}$
BCI IV-2a	ShallowConvNet	$1.843\times 10^{-2}$	$2.701\times 10^{-2}$
BCI IV-2a	ATCNet	$15.03\times 10^{-2}$	$16.15\times 10^{-2}$
BCI IV-2b	EEGNet	$1.507\times 10^{-2}$	$1.927\times 10^{-2}$
BCI IV-2b	ShallowConvNet	$1.296\times 10^{-2}$	$2.533\times 10^{-2}$
BCI IV-2b	ATCNet	$12.58\times 10^{-2}$	$13.51\times 10^{-2}$

## Data Availability

The BCI Competition IV 2a and 2b datasets utilized in this study are publicly accessible. To access it, you can download from the following website: http://bnci-horizon-2020.eu/database/data-sets (accessed on 11 February 2026).
